# Editorial: Insights in interventions for rehabilitation: 2023

**DOI:** 10.3389/fresc.2023.1326850

**Published:** 2023-11-17

**Authors:** Jack Jiaqi Zhang, Ann Van de Winckel

**Affiliations:** ^1^ Department of Rehabilitation Sciences, The Hong Kong Polytechnic University, Hung Hom, Hong Kong SAR, China; ^2^ Division of Physical Therapy, Division of Rehabilitation Science, Department of Rehabilitation Medicine, Medical School, University of Minnesota, Minneapolis, MN, United States

**Keywords:** rehabilitation interventions, editorial, innovative technologies, assessments, digital technology, complementary and integrative practices

**Editorial on the Research Topic** Insights in interventions for rehabilitation: 2023

With the rapid advancement of innovative technologies and evolving theories of functional recovery, the field of rehabilitation interventions has witnessed remarkable progress. We are now experiencing a greater shift toward evidence-based, theory-driven, technology-assisted, remote, or novel in-person rehabilitation interventions. This paradigm shift has the potential to lead to improved recovery outcomes and better quality of life for patients living with disabilities, sequelae of injuries, and various medical conditions.

In recent years, clinical rehabilitation research has investigated several innovative technologies or applied existing technologies and treatments to novel situations. These include home-based digital technology and/or wearable devices (1, 2); home-based non-invasive brain stimulation (3); remote interventions, including complementary and integrative methods (4, 5); virtual/augmented/mixed reality (6); robot-assisted therapy for patients with disabilities from neurological or other origins (7); and brain-computer interface that could be combined with other technologies, such as functional electrical stimulation (8). Furthermore, evidence-based recovery models can guide the implementation of technology into conventional rehabilitation programs (9). Overall, the addition of innovative technologies, especially apps and wearable devices such as smartwatches (10), is revolutionizing the field of (tele)rehabilitation interventions, paving the road for larger and more objective database collection and possibly leading to more personalized rehabilitation.

We are pleased to announce the publication of our Research Topic titled “Interventions for Rehabilitation 2023”, published in *Frontiers in Rehabilitation Sciences*. This collection represents a compact compilation of new insights, recent advances, and future perspectives in the field of rehabilitation interventions. Over the past year, our Research Topic accepted five articles that cover a range of interventional approaches and rehabilitation topics, such as a new app-supported executive function training for adults with traumatic brain injury or post-traumatic stress disease; correlation of self-rated balance ratings with kinematic data from wearable research-grade posturographic sensors, an explanatory theory for implementation of research, a new method of non-invasive brain stimulation, and electro-acupuncture for enhanced recovery after surgery. We believe that this Research Topic provides a sample of the latest developments in rehabilitation interventions.

Shiba et al. conducted a case study exploring the therapeutic mechanisms of bihemispheric transcranial direct current stimulation (tDCS) on improving distal upper limb function in a patient with subacute stroke. The study showed greater improvements in upper extremity function, corticospinal tract excitability, and muscle activity patterns after bihemispheric tDCS compared to anodal tDCS. The case study exemplifies the integration of interhemispheric mechanisms post-stroke and neuromodulatory technology into clinical research.

Waid-Ebbs et al. focused on an executive training protocol (i.e., Goal Management Training), in Veterans with chronic mild traumatic brain injury and post-traumatic stress disorder. The authors complemented ten in-personal training sessions with a Veteran's Task Manager, a Smartphone application. After the training, participants experienced significant improvements in executive function and community participation. The study demonstrated the feasibility of using digital technology for remote rehabilitation, in addition to therapist-led rehabilitation interventions.

Bulley et al. conducted a qualitative analysis of the PEDAL (PrEscription of intraDialytic exercise to improve quAlity of Life in patients with chronic kidney disease) trial, addressing questions relating to trial feasibility and long-term intervention implementation. They interviewed research participants enrolled in the PEDAL trial as well as health care and research staff. The in-depth thematic analysis led to a novel explanatory theory with relevance for evaluation of rehabilitation interventions, elaborating on complex interactions between different aspects of intervention delivery and trial implementation within the clinical environment.

Ferris et al. identified the relationship between physical therapists' ratings of balance intensity (i.e., how well patients can keep their balance), patients' self-assessments of balance intensity, and quantitative posturographic measures when older adults or participants with vestibular disorder-related balance concerns performed standing balance exercises. The results suggested that patients can more easily distinguish two levels of balance ability (higher-lower) instead of five levels of balance ability during their self-ratings; and that self-ratings and trunk kinematic measurements using inertial measurement units may assist in the physical therapists' assessments of the patients' balance performance, especially when visual assessment is difficult (e.g., during telerehabilitation).

Mao et al. provided a review of the potential value and feasibility of electro-acupuncture (EA) for enhanced recovery after surgery. Enhanced recovery refers to a series of optimal perioperative interventions to reduce psychological and physical traumatic stress in order to facilitate rapid recovery of patients, reduce the risk of readmission and death, and decrease costs. EA can attenuate the inflammatory response and stress response, ease anxiety and depression, protect cardiovascular and cerebrovascular function, help postoperative intestinal function recovery, and shorten hospital stay, thereby improving perioperative efficacy. In sum, this review provides evidence-based data to support the notion that EA can play an increasingly important role in rehabilitation and treatment in the future.

## Conclusion

The articles published in this Research Topic describe a small sample of recent developments of novel interventions targeting motor function, cognition, balance, as well as general recovery in patients living with subacute stroke, chronic mild traumatic brain injury, and post-traumatic stress disorder, chronic kidney disease, older adults, adults with vestibular disorder-related balance concerns, or who undergo surgery. The articles highlight the integration of various technologies, including new ways of using non-invasive brain stimulation, digital technology, and complementary and integrative practices, as part of rehabilitation assessments or as interventions. Moreover, one article highlights the use of qualitative methods to reflect on trial feasibility and implementation of interventions.

In sum, despite the small number of published papers, this Research Topic encompasses a heterogeneous sample of new insights, novel developments, current challenges, latest discoveries, recent advances, and future perspectives in the field of interventions for rehabilitation. As this is an ever-evolving field, we hope that this Research Topic will engender new research ideas and collaborations leading to further developments and discoveries in the field of rehabilitation interventions and assessments.
